# A Systematic Review on the Epidemiological Data of Erythema Nodosum Leprosum, a Type 2 Leprosy Reaction

**DOI:** 10.1371/journal.pntd.0002440

**Published:** 2013-10-03

**Authors:** Carlijn G. N. Voorend, Erik B. Post

**Affiliations:** The Royal Tropical Institute (KIT Health), Amsterdam, The Netherlands; University of California San Diego School of Medicine, United States of America

## Abstract

**Background:**

Erythema Nodosum Leprosum (ENL) is a humoral immunological response in leprosy that leads to inflammatory skin nodules which may result in nerve and organ damage, and may occur years after antibiotic treatment. Multiple episodes are frequent and suppression requires high doses of immunosuppressive drugs. Global occurrence is unknown.

**Methodology/Principal Findings:**

Systematic review of evidence on ENL incidence resulted in 65 papers, predominantly from India (24) and Brazil (9), and inclusive of four reviews. Average incidences are based on cumulative incidence and size of study populations (n>100). In field-based studies 653/54,737 (1.2%) of all leprosy cases, 194/4,279 (4.5%) of MB cases, and 86/560 (15.4%) of LL cases develop ENL. Some studies found a range of 1–8 per 100 person-years-at-risk (PYAR) amongst MB cases. Hospital samples indicate that 2,393/17,513 (13.7%) of MB cases develop ENL. Regional differences could not be confirmed. Multiple ENL episodes occurred in 39 to 77% of ENL patients, with an average of 2.6. Some studies find a peak in ENL incidence in the first year of treatment, others during the second and third year after starting MDT. The main risk factor for ENL is a high bacteriological index.

**Conclusions/Significance:**

Few studies reported on ENL as a primary outcome, and definitions of ENL differed between studies. Although, in this review averages are presented, accurate data on global and regional ENL incidence is lacking. Large prospective studies or accurate surveillance data would be required to clarify this. Health staff needs to be aware of late reactions, as new ENL may develop as late as five years after MDT completion, and reoccurrences up to 8 years afterwards.

## Author Summary

This systematic review addresses an underpublicized and yet highly significant leprosy topic. Erythema Nodosum Leprosum (ENL) is a serious complication in multi-bacillary (MB) leprosy that may lead to severe disability. Inflammatory skin nodules may result in nerve and organ damage and require high doses of immunosuppressive drugs. ENL can occur long after patients are released from antibiotic treatment. Frequency and severity of ENL is unknown; this review confirms the lack of accurate data at global, regional, and national levels. Available data indicates that ENL incidence ranges between 0.7–4.6% of all MB cases and late reoccurrence up to 8 years after release from treatment. ENL episodes often reoccur, with an average of 2.6 times. The main risk factor for ENL is a high bacteriological index. Additionally, data indicate a wide variation of ENL occurrence between and within countries. The conclusions demonstrate the need for increased awareness about ENL, in research, patient surveillance, and in programme management.

## Introduction

Erythema Nodosum Leprosum (ENL), the main symptom of a type-2 reaction in leprosy, is caused by a humoral immune response to Mycobacterium Leprae [1]. Patients develop fever and tender/painful subcutaneous nodules, often in the face or extensor surfaces of the limbs [2]–[4]. ENL may also damage nerves, skin, eyes, and testes, and involves systemic illness including fever, weight loss and pain [5], all of which result in extreme discomfort. The majority of patients develop multiple episodes of ENL. Severe cases require the use of potent immunosuppressants, and the steroid-induced side effects may increase mortality and morbidity [3], [6]. Furthermore, the limited use of teratogenic thalidomide presents another challenge [5]. The economic impact of ENL is unknown, but likely to be considerable.

ENL is confined to leprosy patients classified as BL or LL (Ridley-Jopling), comprising the multi-bacillary (MB) patient group, as defined by WHO. In 1981 this concerned patients with a bacteriological index (BI) of 2 or more, changing to any positive skin smear in 1988. In 1995 this was widened further; MB comprising any patients with more than five skin lesions [7]. The proportion of MB cases among new leprosy patients varies between countries and is increasing [8], [9]. Global incidence of MB leprosy was 139,125 in 2009, and is decreasing [8]. ENL may occur before, during or after antibiotic treatment, and several years later [10]. It can occur as a single acute episode, but frequently develops into a chronic condition with recurrent episodes [3], [5]. Immune responses causing ENL are triggered by high loads of fragmented bacilli in skin tissue [11].

Although adequate surveillance systems are used to estimate global leprosy prevalence and inform drug supply, this is not available for estimating incidence, frequency and severity of ENL [12]. Geographic variation in ENL prevalence complicates accurate estimations [13], and hampers logistics in drug supplies. For this reason, a systematic literature review was conducted to determine global incidence of ENL, inclusive of incidences of recurrent and severe ENL and contributing factors.

## Methods

### Searching

A systematic literature search was conducted in January 2011 in five databases (Pubmed (MEDLINE), EMBASE, LILACS, SCOPUS, Scielo, and Ajol). Keywords used were: <lepro* OR lepra* OR hansen*, Erythema Nodosum OR ENL OR (type 2)>, AND <incidence OR prevalence OR cohort>. Reference lists of included studies were checked and national leprosy control managers and leading leprologists were asked for additional (un-)published articles.

### Inclusion criteria

Studies, published after 1980, presenting data on incidence or prevalence of ENL were selected. Focus was on papers in English, whereas Portuguese, Spanish or French studies were included after Google-translation. No separate search was conducted on adverse events and risk factors, but information was retrieved from the included studies. A distinction was made between acute and chronic ENL as well as severe and mild forms [2]. We included all studies reporting on the onset of ENL. The following forms of ENL were included: single acute episodes, multiple acute episodes, and chronic ENL (ENL lasting for more than 6 months, in either single or multiple episodes) [2].

### Data synthesis and analysis

Data extraction regarding onset, risk factors, severity and reoccurrence of ENL was completed by the first author and co-reviewed by the second author. A structured form was designed to retrieve data on the setting (country, region, place studied, other characteristics), methods (study period, design, sampling, data sources, representativeness), study design and characteristics (sample size, population, leprosy classification (Ridley-Jopling), inclusion criteria, ethnicity, gender, age group, other (health) characteristics and study variables (follow-up time, loss to follow up, and MDT-, ENL-, or other treatment, serious adverse events). Evidence was graded according to the Oxford Centre for Evidence Based Medicine guidelines [14].

Depending on availability, incidence rates of ENL are presented in person years at risk (PYAR). Where proportions or actual numbers of patients developing ENL were reported, ENL incidence is based on the proportion of persons at risk (i.e. total number of leprosy cases, MB cases or specific Ridley-Jopling classifications). We considered cases MB as reported in the articles. Occurrence is only presented when sample sizes exceeded 100 at risk (MB) population, for field and hospital studies separately. The average incidence of ENL was calculated taking all different sample sizes together.

## Results

### Trial flow and study characteristics

**Figure 1:**
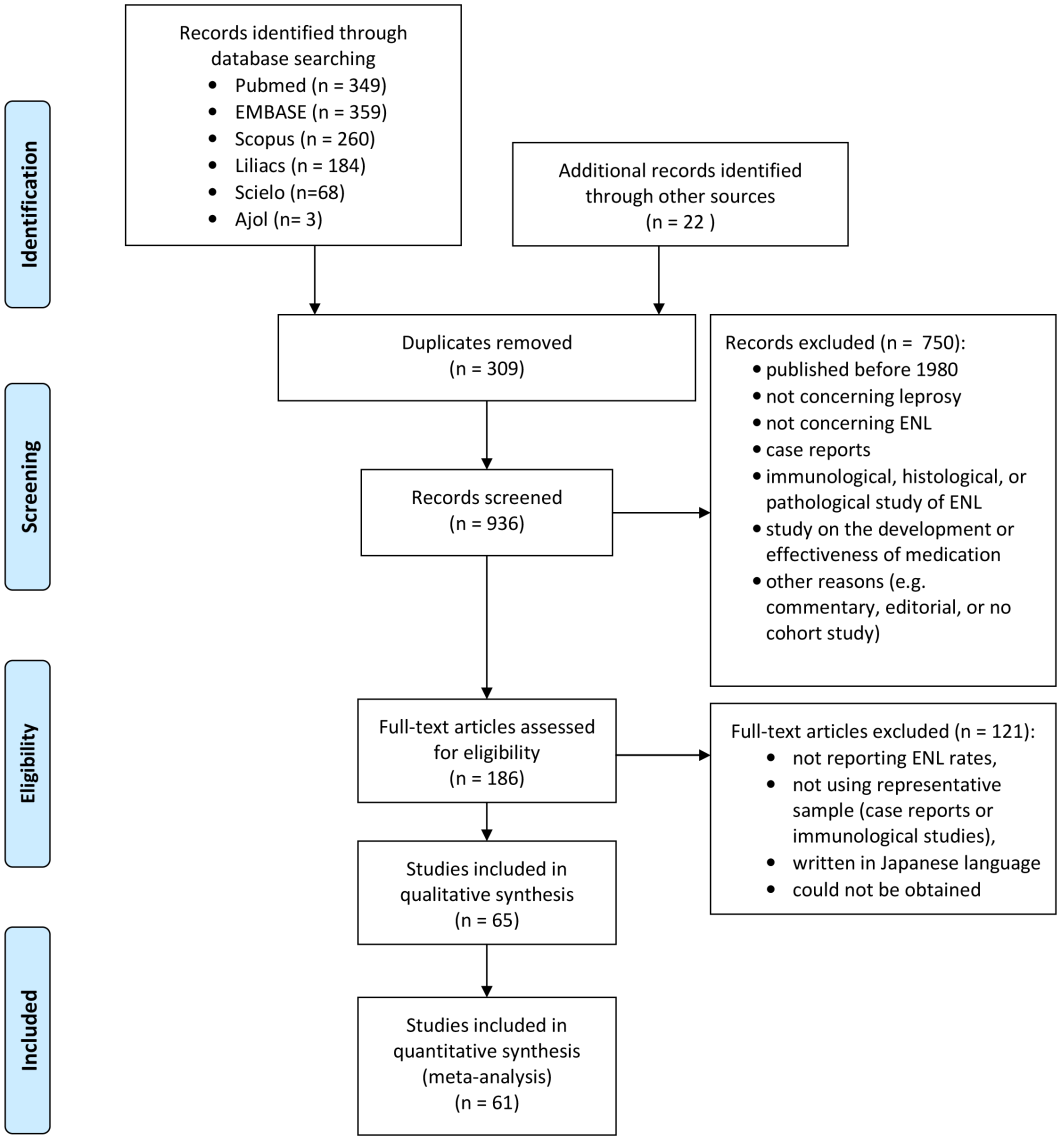
Flow diagram of included studies.

The search resulted in 914 records (Figure 1). Scanning the references and consultation with experts resulted in an additional 10 papers. 65 papers met the inclusion criteria. Four literature reviews were analysed separately [2], [12], [15], [16]. One relevant workshop report was included [17].

The majority of studies were from India (24) and Brazil (9), the two countries with the highest incidence of new leprosy cases [8]. Table 1 summarises the characteristics of included studies. Approximately one third of the studies included a minimum of 300 persons at risk for ENL and another third between 100 and 300 persons. 23 studies had sample sizes below 100 persons at risk [10], [18]–[39]. Studies were either cross-sectional or retrospective cohort analyses. Less than half of them aimed specifically at ENL occurrence. The majority reported ENL frequency while their main focus was on clinical or epidemiological aspects of leprosy.

**Table 1:** Characteristics of included studies (n = 61).

Study characteristic		n (%)
Country	Africa (incl. Middle East)	10 (16)
	India	24 (39)
	Asia (other)	11 (18)
	Latin America	10 (16)
	Developed countries	6 (10)
Study design	Observational cohort (prospective)	13 (21)
	Observational cohort (retrospective)	13 (21)
	Cross-sectional sample	24 (39)
	Controlled trial	9 (15)
	Other	2 (3)
Main aim of study	Occurrence or risk factors of reactions	26 (43)
	Effect of vaccine or treatment regime	11 (18)
	Clinical or epidemiological patterns of leprosy	10 (16)
	Other†	14 (23)
Place studied/reported	Field study	10 (16)
	Medical facility (often tertiary)	50 (82)
	Both field and hospital*	1 (2)
Study sample	Leprosy patients	35 (57)
	MB or lepromatous only	17 (28)
	Other selection‡	9 (15)
Number of at risk cases	MB or lepromatous, n>300	20 (33)
	MB or lepromatous, n = 100–300	19 (31)
	MB or lepromatous, n<100	18 (30)
	Not specified	4 (7)

* Different data sources pulled together at a workshop of the Indian Association of Leprologists.

† Main aim concerned e.g. disability, renal disease, nerve function impairment, or drug regimen.

‡ Study sampled of e.g. discharged, passed away, or leprosy patients with a history of reactions.

### Incidence in person years at risk

**Figure 2:**
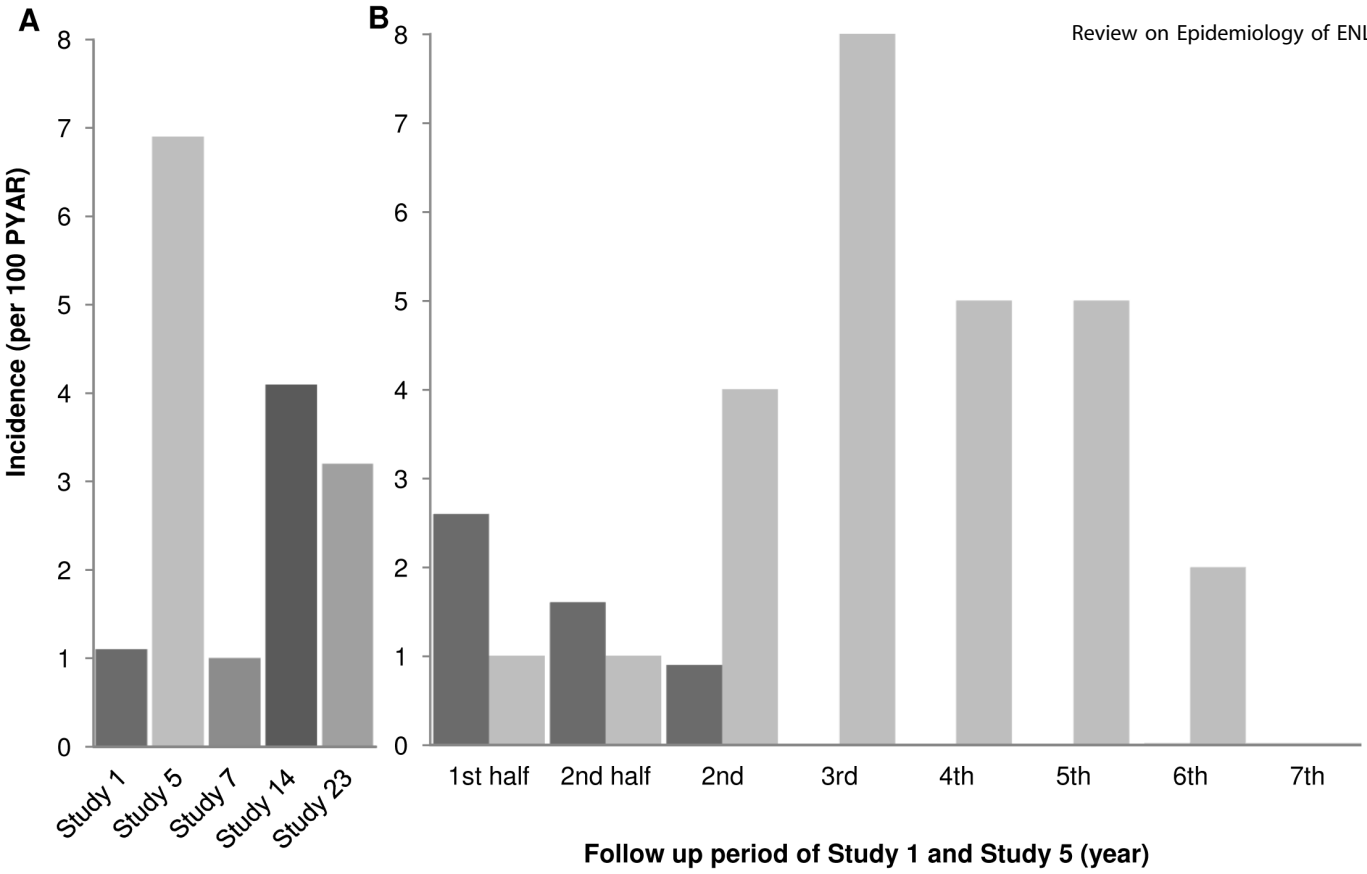
Incidence of ENL reported per person years at risk. (A) Incidence for studies reporting incidence per 100 PYAR. (B) Incidence over time during different study periods for a Bangladesh [43] and Ethiopian [41] study.

Only five studies reported ENL incidence rates in person years at risk (PYAR). Follow up varied between 2 and 7 years. Incidence rates ranged from 1 to 8 per 100 PYAR [40], [41] among MB leprosy patients (figure 2).

### ENL in field leprosy control programmes

Six prospective [30], [41]–[45] and five retrospective studies [17], [40], [46]–[48] gathered data from a control programme and most accurately reflected ENL occurrence.

Table 2 demonstrates that cumulative ENL incidence varied from 0.2% among all leprosy patients in an Indian study [49] and up to 4.6% in a Chinese study [48], with an average of 1.2%. ENL incidence among MB cases varied from 1.0% in a one year cross-sectional Indonesian study [46] to 8.9% in an Indian cohort [47], with an average of 4.5%. From the latter study, it was not clear if referral cases were included, which may explain the relatively high percentage. Three prospective studies were from the ALERT leprosy control services [41], [42], [45]. Interestingly, cumulative ENL incidence was 2.5% among MB cases after an average follow-up of 2.5 years [45], whereas after 10 years this was doubled [41].

**Table 2:** Incidence of ENL in field based studies (n>100).

Study number	Country	Level of evidence	Study design	Follow up period	*Leprosy cases*		*Study sample at risk*		
				years	n	ENL (%)*	n	definition	ENL (%)*
1	Bangladesh [43]	1b	Observational cohort (prospective)	5	2,510	8 (0,3)	357	MB†	8 (2,2)
2	Thailand [44]	2b	Observational cohort (prospective)	>2	640	16 (2,5)	133	BL+LL	16 (12.0)
3	Ethiopia [42]	2b	Observational cohort (prospective)	3.5	-	-	375	BL+LL†	19 (5)
4	Ethiopia [45]	2b	Observational cohort (prospective)	mean 2.5, max 4	286	4 (1.4)	158	MB†	4 (2.5)
5	Ethiopia [41]	2b	Observational cohort (prospective)	max 10	594	16 (2.7)	300	MB†	16 (5.3)
6	India [49]	2b	Observational cohort (prospective)	<1 to 7	2,053	4 (0.2)	106	MB†	4 (3.8)
7	Bangladesh [40]	2b	Observational cohort (retrospective)	unknown	786	10 (1.3)	471	MB	10 (2.1)
8	India [47]	2b	Observational cohort (retrospective)	7	13,465	95 (0.7)	1,067	BL+LL†	95 (8.9)
9	China [48]	4	Cross-sectional	n/a	6,393	294 (4.6)		†	
10	Indonesia [46]	4	Cross-sectional	n/a	856	9 (1.1)	726	MB	9 (1.2)
					751	13 (1.7)	586	MB	13(2.2)
11	India [17]	5	Collected data	unknown	26,403	184 (0.7)	-	-	-
	**Average incidence**				**54,737**	**653 (1.2)**	**4,279**		**194 (4.5)**

* It should be noted here that cumulative incidence is presented as these have been published, although not all numbers could be traced and justified after conducting calculations while some inconsistencies were noticed. So therefore, these numbers should be treated with caution.

† Studies that conducted slit skin smears. Studies not indicated with this footnote did not provide information on conducting slit skin smears.

### ENL in hospital settings

Table 3 indicates the cumulative ENL incidence in 28 studies (>300 patients), ranging from 2–28.9% of MB cases. Calculation from studies with at least 100 patients reveals that on average 13.7% of MB cases developed ENL. In four studies this was more than 30% [50]–[53]. Studies with largest population sizes indicated lower cumulative incidence rates.

**Table 3:** Incidence of ENL in hospital populations (n>100).

Study number	Country	Level of evidence	Study design	Follow up period	Study sample at risk		
				years	N	definition	ENL (%)*
12	India [73]	1b	Observational cohort (prospective)	2	303	MB†	6 (2)
13	India [74]	1b	Observational cohort (prospective)	<8	980	MB†	2 (0.2)‡
14	Thailand [50]	1b	Observational cohort (prospective)	3	119	BL+LL†	44 (37)
15	Thailand, Philippines, Korea [75]	2b	Controlled trial	5	358	BL+LL†	36 (10)
16	India [55]	2b	Controlled trial	8	304	BB+BL+LL†	30 (10)
17	India [76]	2b	Observational cohort (retrospective)	>2–10	578	BB+BL+LL†	164 (28.4)
18	India [66]	2b	Observational cohort (prospective)	6	100	MB†	6 (6.0)
19	India [60]	2b	Observational cohort (retrospective)	>1	481	BL+LL†	117 (24.4)
20	Philippines [63]	2b	Observational cohort (retrospective)	4	296	MB†	36 (12.2)
					293	MB†	60 (20.5)
21	Philippines [61]	2b	Observational cohort (prospective)	3	139	MB†	10 (7)
					295	MB†	27 (9)
22	Zaire [59]	2b	Controlled trial	3	280	MB†	34 (12)
23	Nepal [62]	2b	Observational cohort (retrospective)	2	175	BL+LL†	10 (5.7)
24	Brazil [77]	2b	Observational cohort (retrospective)	2	169	BB+BL+LL†	43 (25.4)
25	Brazil [51]	2b	Controlled trial	2	140	MB†	48 (34.2)
26	Brazil [52]	2b	Observational cohort (retrospective)	2	162	BB+BL+LL†	51 (31)
27	Uganda [56]	4	Cross-sectional	5	2,743	MB	18 (0.7)§
28	India [64]	4	Observational cohort (retrospective)	>2	990	BB+BL+LL†	121 (12.2)
29	India [69]	4	Cross-sectional	1	1141	MB†	187 (16.4)
				1	1,344	MB†	235 (17.5)
30	India [58]	4	Observational cohort (retrospective)	3–13	1,494	MB†	337 (22.5)
31	Nepal [57]	4	Cross-sectional	unknown	563	BL+LL†	107 (19)
32	Brazil [67]	4	Cross-sectional	Unknown	664	MB†	192 (28.9)
33	Netherlands [68]	4	Cross-sectional	Unknown	231	BB+BL+LL	17 (7.4)
34	Morocco [53]	4	Cross-sectional	Unknown	229	MB	76 (33)
35	Brazil [65]	4	Observational cohort (retrospective)	Unknown	218	MB	28 (13)
36	India [54]	4	Cross-sectional	2	187	BB+BL+LL†	25 (13.3)
37	Yemen [78]	4	Cross-sectional	unknown	123	BB+BL+LL†	33 (26.8)
38	Brazil [79]	4	Cross-sectional	unknown	120	MB†	13 (10.8)
11	India [17]	5	Collected data	-	6,017	Leprosy	301 (5)
	**Average incidence (n>100)**				**17,513**		**2,393 (13.7)**

* It should be noted here that cumulative incidence is presented as these have been published, although not all numbers could be traced and justified after conducting calculations while some inconsistencies were noticed. So therefore, these numbers should be treated with caution.

† Studies that conducted slit skin smears. Studies not indicated with this footnote did not provide information on conducting slit skin smears.

‡ Assessed late leprosy reaction during surveillance that started after MB-MDT course until smear negativity. This study is excluded from the calculations.

§ Assessed admissions due to leprosy reactions. This study is excluded from the calculations because n is not well defined.

### ENL for different Ridley-Jopling classification

**Figure 3:**
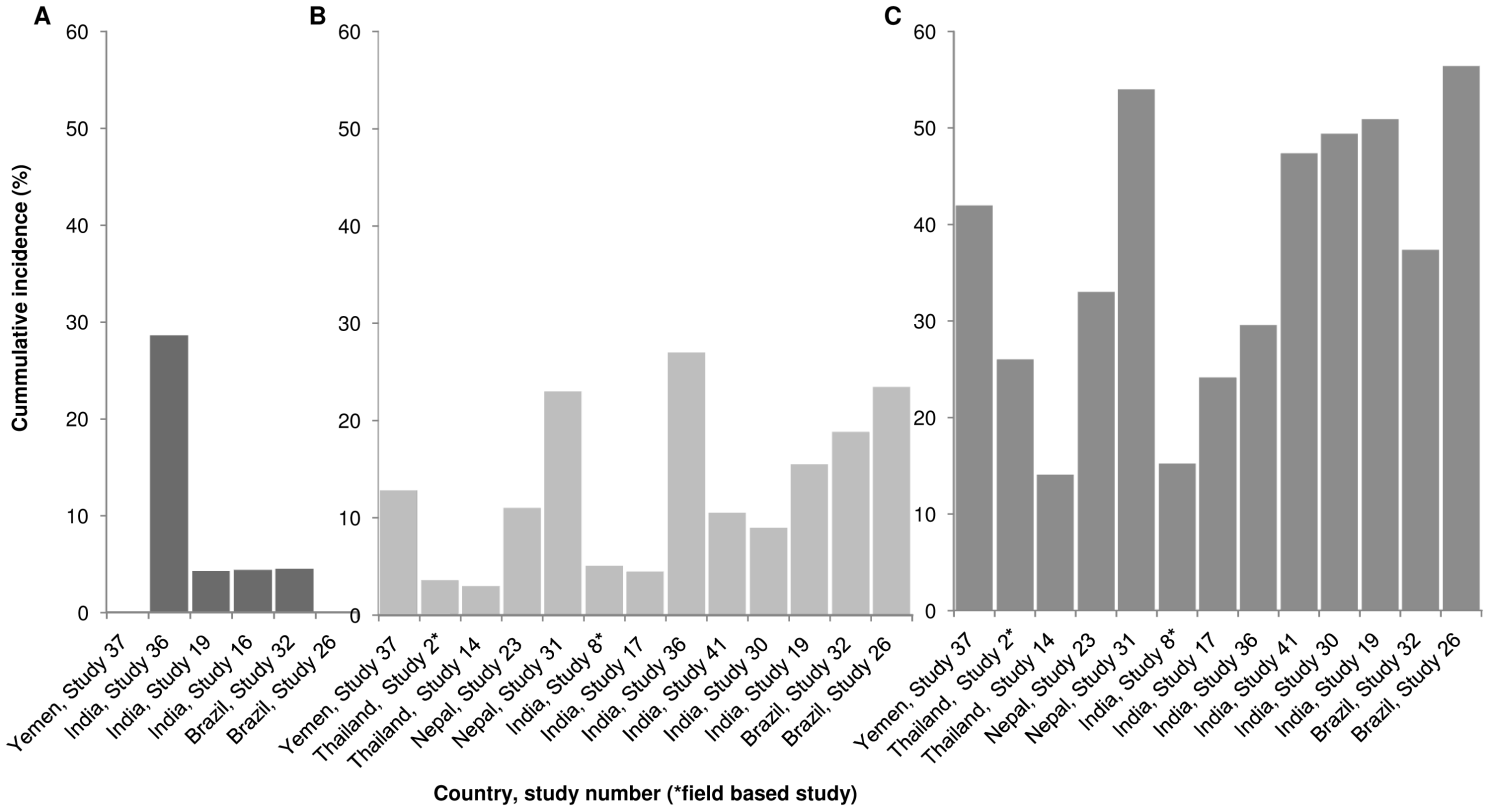
Variation in proportion of cases developing ENL. (A) Incidence (%) for studies reporting for BB cases. (B) Idem for BL cases and (C) LL cases.

Sixteen studies reported ENL occurrence for the Ridley-Jopling classifications (Figure 3). Findings differed widely between countries. Among the four field studies [41], [42], [44], [47] ENL for LL leprosy ranged from 11.1% [42] to 26% [44] with an average of 86/560 (15,4%). For BL cases this varied from 2.7% [42] to 5.1% [47], on average 51/1231 (4,1%). In hospital based studies higher proportions were found, in Brazil up to 56.4% [52] and in India a range of 24.2 [54] to 50.9% [55].

### Multiple episodes of ENL

ENL reoccurrence was disproportionately higher in hospital-based studies. Multiple episodes were found in 39% [56] to 77.3% [50] of ENL patients, with an average of 2.6 episodes. Various studies reported 24% of all ENL cases having more than four episodes: the longer the follow-up the more episodes were recorded. Three larger studies (>100 ENL cases, see Table 4) found a range from 49% [57] to 64.3% [58]. Similar ranges were found in field based studies: 44 to 63% of all ENL cases have multiple ENL episodes [41], [44], [45].

**Table 4:** Findings on multiple episodes, number and duration of ENL episodes.

Study number	Country	Type of study	LE	Study design	Follow up/study period (years)	ENL cases	ENL cases with >2 episodes (%)	Average number of episodes	Average duration episode (weeks)
2	Thailand [44]	Field	2b	Observational cohort (prospective)	<2	16	7 (44)		
4	Ethiopia [45]	Field	2b	Observational cohort (prospective)	mean 2.5, <4	4	2 (50)	(range 1–3)	
5	Ethiopia [41]	Field	2b	Observational cohort (prospective)	<10	16	10 (63)	3 (range 1–8)***	
8	India [47]	Field	2b	Observational cohort (retrospective)	5 after RFT	92	47 (51.1§)		
9	China [48]	Field	4	Observational cohort (retrospective)	1	293		2.9	
14	Thailand [50]	Hospital	1b	Observational cohort (prospective)	<3	44	34 (77.3)	“often >4 times”	
16	India [55]	Hospital	2b	Controlled trial	mean 8.5- 8.4	50	26 (52)		
16a						51	30 (58.8)		
17	India [76]	Hospital	2b	Observational cohort (retrospective)	<4	164	164 (†)		
19	India [60]	Hospital	2b	Observational cohort (retrospective)	>1	88**	81 (92††)	3.2 (CI 2.7–3.5)	
20	Philippines [63]	Hospital	2b	Observational cohort (retrospective)	4	60		2.9	5.3
20a						36		2.4	17
21	Philippines [61]	Hospital	2b	Observational cohort (prospective)	2 after RFT	8			15
21a						10			26.1
22	Zaire [59]	Hospital	2b	Controlled trial	<3	34		1.8	8.5
26	Brazil [52]	Hospital	2b	Observational cohort (retrospective)	2	51	38 (74.5‡‡)	2.5	
27	Uganda [56]	Hospital	4	Cross-sectional	5	18	7 (39)	1.4	
30	India [58]	Hospital	4	Observational cohort (retrospective)	3–13	337	217 (64.3)	2.6	
31	Nepal [57]	Hospital	4	Cross-sectional	8	107	52 (49‡)	2	
39	India [18]	Hospital	3b	Controlled trial	<4	10	7 (70§§)	2.0	
39a						12	9 (75§§)	2.4	
40	India [19]	Hospital	4	Cross-sectional	<1	17			14.35 days (sd 3.53; range 8–20)
	**Total**					**896**	**567 (63.3)**	**2.6**	

† Only cases with multiple episodes of ENL reported, this accounted for 28.4% of MB cases. This study is excluded from the calculations.

‡ 49 (45%)single, 27 (25%) two, 13 (12%) three, 6 (5%)four, 2 (2%) five, 5 (5%) >five episodes.

§ 45(49%) single, 28 (30%)two, 14 (11%) three, 5 (5%) four or more episodes.

** Of the original cohort of 116 patients, 28 were excluded because they had too short follow-up and could not be categorized.

†† 37.5% having acute multiple ENL (i.e. more than one episode lasting less than six months, steady decrease in steroid tapering) and 62.5% chronic ENL (i.e. episode lasting for more than six months).

‡‡ 13 (25%)single, 12 (24%)two, 14 (27%)three, 11(22%)four, 1(2%)five episodes.

§§ Vaccine versus control group; 3 vs 3 single, 4 vs 4 two, 3 vs 2 three, 0 vs 3 more than three episodes.

*** An episode of ENL was taken as a separate event if more than 3 months had elapsed since the last episode.

There was discrepancy in the average number of ENL episodes, as is evident in the following findings. In a cohort from Zaire [59] there was an average of 1.8 episodes, compared to 3.2 episodes (CI 2.7–3.5), in a study from India [60]. A Thai cohort revealed that ENL episodes often occurred more than 4 times [50]. A large hospital study in India reported that 23.5% of reoccurring cases (15.1% of all ENL cases) had four or more episodes [58]. Similar proportions were found in a Brazilian cohort [52], whereas other studies in India [47] and Nepal [57] found four or more episodes among 5 and 7% of ENL patients respectively. In Ethiopia, almost one third of ENL patients developed a chronic condition lasting more than 2 years [41]. Episodes lasted from 14 days [19] to 26.1 weeks [61]. Total ENL episodes and ENL-free intervals in India found an average of 18.5 months (CI 15.4–21.5) [60].

### Severity

Six studies distinguished between mild and severe ENL, finding that 30–50% of ENL cases are (moderate to) severe. They represented 0.7–2.0% of all MB leprosy patients and 0.7% of all newly detected cases [46], [62]. However, descriptions of severity differed between the studies. Shortened MDT duration (12 months) almost doubled the incidence of moderate to severe ENL [61], [63]. Poor referral practices leave some severe reactions under-diagnosed [40], while hospital figures misrepresent the field situation [47].

### Onset of ENL in relation to MDT

Findings on the onset of ENL differ. Most studies indicated that the incidence of ENL during MDT was at least twice as high than at the time of the initial diagnosis [37], [42], [44], [50], [64], [65]. ENL incidence was highest in the first year of MDT [17], [37], [42], [44], [57], [58], [64]. There were a few exceptions, a from the Philippines (10 year follow-up) [43], [61] and India (13 years follow-up) [58] where most ENL was diagnosed during the second and third year after starting MDT, as was the case in Ethiopia [41].

A study conducted in an Indian hospital found 3% of MB patients developed ENL two years after completing MDT (follow-up 74 months) [58]. Longer term follow up showed ENL three [37], five [66], seven [41], or even eight years after MDT [58]. Similar findings (ENL occurring 5–7 years later) were reported in India [17].

### Contributing risk factors to the development of ENL

Multiple studies [22], [23], [52], [57], [58], [60], [62] reported a correlation between the bacteriological index (BI) and ENL up to a 8.6 (CI 2.3–32) times higher risk when having a BI of six [41]. Discrepancies are evident Nepali patients with a BI>4+ had a 39% higher risk of ENL (OR; 1.39 (CI 1.11–1.76) adjusted for age) [57] and in India a BI≥4 was associated with an Odds Ratio of 5.2 (2.1–12.9) [60]. Inherent to BI, lepromatous leprosy is a significant risk factor [58], [67]. An Ethiopian study found a 9.6 times higher ENL incidence among LL patients compared to BL or BB (X^2^ = 18.7, p<0.005) [42]. Odds ratios for the prevalence of ENL in LL as compared to BL varied from 2.8 (1.59–5.2; adjusted for age and BI) [57] to 8.4 (CI 4.6–15.4) [60]. LL cases have higher chances to suffer multiple rather than single ENL episodes (OR 2.94, p = 0.052) [57]. This finding was disputed, however, by a controlled clinical trial conducted in India, which reported no such differences [55].

It has been claimed that the risk of developing ENL has decreased since introducing MDT [42], [51], [54], [57], due to the ENL suppressant effect of clofazimine [22], [51], [68]. A recent multi-country cohort study indicated more severe and longer-lasting episodes of ENL among patients who received 12 as compared to 24 months of MDT, although ENL frequency as such was similar [61], [63].The Bombay Leprosy Project had similar findings: 55.9% and 35.8% of cases receiving 12 and 24 months MDT respectively had a type1 or 2 reaction [17].

Gender is generally not a risk factor for ENL [41], [52], [55], [57], [60], [62], [63]. Some studies appear to challenge this, as a large hospital study in India found a male predominance [69], and a large Indian cohort reported a higher risk for women [58]. These differences, however, may be due to differences in health seeking behaviour [69].

Seemingly, age is not a risk factor for ENL [41], [50], [58], [60], [63], although a Nepali cohort indicated decreased risk for those older than 40 (adjusted OR 0.69, CI 0.5–0.94) [57], and a higher ENL incidence was seen in patients diagnosed with leprosy in their adolescence, but these findings are not supported elsewhere [50].

Pregnancy and lactation appears to be a significant precipitating factor for severe and recurrent ENL [54]. Additionally, hormonal changes are implicated in a study from India: 62% of 32 ENL in women were associated with pregnancy or lactation and 21% with menopause [69]. A major Ethiopian study among pregnant leprosy patients found an increased ENL incidence (22% among BL and 59% among LL patients). Some episodes continued until 15 months after delivery [24].

Minimal evidence has been published regarding co-morbidities as risk factors for ENL, with the exception of HIV that suggested a 5.3 times higher risk for developing ENL (RR 5.3, CI 1.0–2.8). However, numbers (n = 10) were too low to be conclusive [41]. A recent review concluded there is no reliable data on the effect of HIV [13]. In other studies, malaria and tuberculosis were reported to trigger ENL [24], [54].

## Discussion

Presenting a comprehensive overview of the epidemiological data on ENL incidence, was difficult due to lack of available and reliable data. Furthermore, few studies reported ENL as a primary outcome. Findings were complicated by the inconsistency in case definitions of ENL. Additionally, much of the data drawn on in this review was prior to the WHO-MDT era, asserting that 50% of LL patients and 25% of BL patients developed ENL in the course of the disease [12], [70]. This review establishes that prevalence rates are highly variable, in field cohorts up to 26% LL and 5.1% BL patients, and 37% in a hospital sample of MB patients. In an effort to overcome the difficulty of variations in ENL occurrence, average incidences were calculated in field based populations for all leprosy cases (1.2%) and for MB leprosy cases (4.5%). In hospital samples these percentages were higher. This review could not confirm any regional differences and found differences between and within countries.

Few comprehensive prospective studies reported ENL incidence in terms of person years at risk and controlled for confounding factors. Estimates presented in this paper should therefore be taken with caution. We underline the lack of reliable epidemiological data due to the absence of a universally-accepted set of norms and standardized nomenclature as well as lack of awareness and recording [52]. Standardized definitions should be set globally and would facilitate the collection of better quality data. Well-designed field studies to ascertain this have been called for [71]. All findings considered, the authors are of the opinion that if national estimates are needed (e.g. for estimating local needs for clofazimine to treat severe ENL), this is best done on the basis of local evidence and indications by experienced programme and clinical staff.

Alarmingly, ENL reoccurs, and often more than four times, in almost a half of initial ENL reported episodes. Multiple episodes were found in 39–77.3% of ENL patients. Calculations indicate an average of 2.6 episodes per ENL patient. Episodes of ENL peak during MDT, but also occur up to 7–8 years after release from treatment [65]. Therefore, it is imperative that both patients and health workers are on the alert for development of late episodes of ENL [17], [60]. It is of major concern that leprosy control programmes do usually not advocate standardized follow-up [65].

The main risk factors for developing ENL are related to a high bacteriological index and a BL/LL classification in the Ridley-Jopling spectrum [13]. The ENL-suppressive effect of clofazimine, within the MDT regimen, is generally acknowledged [68]. More severe and longer-lasting ENL episodes occur in shorter duration MDT-course (12 months as opposed to 24).

There was no conclusive evidence for co-morbidities or age as risk factors. Possible precipitating factors for ENL included hormonal changes occurring in pregnancy, lactation, menopause, and puberty. Additional findings suggest that intercurrent infection, vaccination and psychological stress, are implicated (Pfaltzgraff and Ramu in Clinical Leprosy) [70]. This appears to be supported by empirical evidence only, and was not confirmed by this literature review. This may be explained by the lack of large prospective studies and relatively low incidence of ENL and co-morbidities. Perhaps the analysis of large existing data sets (e.g. BANDS, AMFES, INFIR, Brazil, possibly other countries) may help in identifying precipitating factors. Prospective studies would be required to elucidate hormonal and genetic risk factors [12].

### Limitations

Most of the literature regarding ENL occurrence was descriptive data, and only a few studies had an adequate sample of patients. Characteristics of cases and populations, definitions, outcomes and procedures were not always systematically described, making a statistical meta-analysis impossible.

To what extent study samples reflected the leprosy population at large was often difficult to assess, as distinction between field and hospital based studies was not clear in each publication. Higher ENL rates were found in hospital based studies, although it is not known how many severe ENL cases actually arrive in referral clinics. In the hospital based studies the population size of which these cases are drawn is not known. Field based studies often only report patients with ENL who actually seek help. Only few appropriate prospective studies could be found that are representative for the most peripheral level.

The majority of publications lacked both a clear case definition of ENL and a clear description of the diagnostic procedure. Both may vary between settings and studies. Only a few studies make a distinction between mild and severe ENL [60]–[63], and mild ENL may have been overlooked and thus incidence rates underestimated.

Considering the limited evidence and the significant differences in ENL rates, country specific data should be interpreted with great caution. The wide range in cumulative incidence and variation of ENL found in this review is most likely explained in terms of duration of treatment and follow-up of the subjects. Furthermore, the widening definition of MB leprosy since 1981 [4], [72] would have decreased rates of ENL. LL patients would be the most appropriate risk group for ENL to report on, especially in research papers. In this study, however, MB was the most common denominator in the articles that were identified. Ideally, future studies on ENL should report incidence in person years at risk, both for MB and Ridley-Jopling classification.

None of the studies included in this review looked at explicitly at the social and medical costs related to ENL.

### Conclusion

This review provides a systematic overview of available evidence regarding ENL occurrence. Wide ranges were found between and within different countries. Despite these limitations, a global average incidence was calculated. This review has established that reliable data on ENL occurrence is lacking, and could only be obtained through large comprehensive prospective studies or data obtained from accurate ENL surveillance. Furthermore, studies investigating risk and precipitating factors for ENL would be useful in diagnosis and prevention.

## Supporting Information

Supporting Information S1 Prisma statement checklist and flowchart.
(DOC)
